# Dual inhibition of DNA-PKcs and mTOR by CC-115 potently inhibits human renal cell carcinoma cell growth

**DOI:** 10.18632/aging.103847

**Published:** 2020-10-27

**Authors:** Bing Zheng, Xu Sun, Xin-Feng Chen, Zhan Chen, Wei-li Zhu, Hua Zhu, Dong-Hua Gu

**Affiliations:** 1 Department of Urology, The Second Affiliated Hospital of Nantong University, Nantong, China; 2 Department of Hand and Foot Surgery, Hospital Affiliated 5 to Nantong University, Taizhou People’s Hospital, Taizhou, China; 3 Port Clinic, Changshu Customs, Changshu, China

**Keywords:** renal cell carcinoma, DNA-PKcs, mTOR, CC-115, autophagy

## Abstract

CC-115 is a dual inhibitor of DNA-PKcs and mTOR, both are valuable therapeutic targets for renal cell carcinoma (RCC). Our results showed that CC-115 inhibited survival and proliferation of established RCC cell lines (786-O and A489) and primary human RCC cells. The dual inhibitor induced selective apoptosis activation in RCC cells, as compared to no cytotoxicity nor apoptotic effects toward normal renal epithelial cells. CC-115 inhibited DNA-PKcs and mTORC1/2 activation in RCC cells. It was however ineffective in DNA-PKcs-mTOR double knockout (DKO) 786-O cells. CC-115 induced feedback autophagy activation in RCC cells. Autophagy inhibitors or Beclin-1/Light chain 3 (LC3) silencing potentiated CC-115-induced anti-RCC cell activity. Conversely, ectopic overexpression of Beclin-1 inhibited CC-115-induced cytotoxicity. At last CC-115 oral administration inhibited 786-O subcutaneous xenograft growth in nude mice. Taken together, dual inhibition of DNA-PKcs and mTOR by CC-115 potently inhibited RCC cell growth.

## INTRODUCTION

Renal cell carcinoma (RCC) causes significant human mortalities each year [1]. Identification of biomarkers for RCC is important for clinical diagnosis and development of better therapeutic strategies [2]. Mammalian target of rapamycin (mTOR) is the central player of the phosphoinositide 3-kinase (PI3K)-AKT-mTOR cascade [3, 4]. It is frequently overactivated in RCC [5]. mTOR overactivation is due to different mechanisms including loss of function mutations or depletion of PTEN, activating mutations of PIK3CA, or sustained activation of receptor tyrosine kinases (RTKs) [6–8]. Two mTOR complexes, mTOR complex 1 (mTORC1) and mTOR complex 2 (mTORC2), have been identified thus far [8].

Everolimus and other mTORC1 inhibitors are currently utilized for the clinical treatment of certain advanced RCCs [6, 8]. However, mTORC1 inhibitors have several drawbacks. First, mTORC1 inhibitors can only partially inhibit mTORC1 [9, 10]. Second, mTORC1 inhibition will induce feedback activation of pro-cancerous cascades, including the PI3K-Akt and Erk-MAPK signalings [9, 10]. Furthermore, mTORC1 inhibitors are unable to directly block mTORC2 activation [11, 12], the latter is important for RCC progression [7]. Recently, mTOR kinase inhibitors which block both mTORC1 and mTORC2 have been developed [9, 10]. Our previous studies have shown that the mTOR kinase inhibitors can potently inhibit RCC cell growth *in vitro* and *in vivo* [11, 12].

A second valuable therapeutic target of RCC is DNA-activated protein kinase (DNA-PK). It is composed of three primary members, including the catalytic subunit (DNA-PKcs) and Ku hetero-dimer (Ku70/Ku80) [13, 14]. When activated, the 460-kDa DNA-PKcs initiates non-homologous end joining (NHEJ) signaling to repair DNA double-strand breaks [13, 14]. Existing studies, including ours [15], have demonstrated the significant cancer-promoting function of DNA-PKcs. DNA-PKcs is important for AKT-mTORC2 activation, regulating cancer cell survival, proliferation and resistance to radiation/chemotherapy [16–18].

Our previous study has shown that DNA-PKcs levels are elevated in RCC tissues and cells, important for RCC cell growth [15]. DNA-PKcs inhibition or silencing potently inhibited RCC cell progression [15]. DNA-PKcs interacted with the mTORC2 complex in RCC cells to mediate AKT activation (Ser-473 phosphorylation) and hypoxia-inducible factor-2α (HIF-2α) expression [15]. Therefore, targeting DNA-PKcs is a valid strategy to inhibit RCC cell growth.

A recent study by Mortensen et al., has characterized a DNA-PKcs/mTOR dual inhibitor CC-115 [19]. The orally active dual inhibitor blocks DNA-PKcs and mTORC1/2 signaling [19, 20]. CC-115 displayed favorable physicochemical and pharmacokinetic properties along with acceptable safety profiles [19, 20]. It is therefore suitable for potential clinical development [19]. Here we examined the efficacy of CC-115 against RCC cells.

## RESULTS

### CC-115 inhibits human RCC cell survival and proliferation

**Figure 1:**
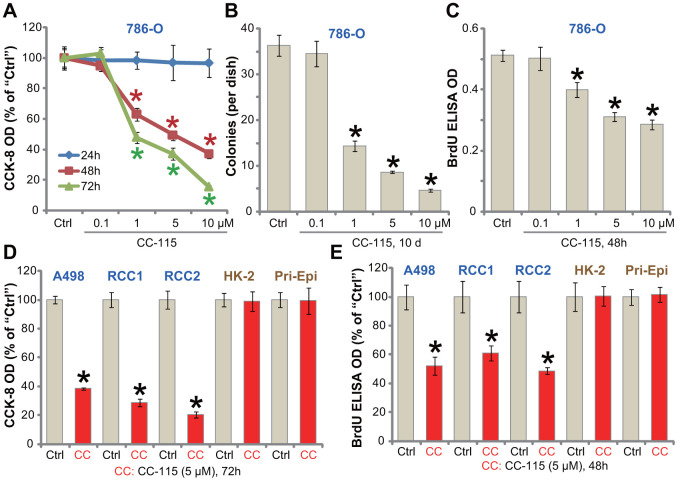
**CC-115 inhibits human RCC cell survival and proliferation.** Established human RCC cell lines (786-O and A498), the primary human RCC cells (derived from two patients, “RCC1/2”), immortalized HK-2 tubular epithelial cells as well as the primary human renal epithelial cells (“Pri-Epi”) were treated with indicated concentration of CC-115 for the applied time periods, cell viability was tested by CCK-8 assay (**A**, **D**); Cell proliferation was tested by soft agar colony formation assay (**B**) and the BrdU ELISA assay (**C**, **E**). Data were expressed as mean ± standard deviation (SD, same for all Figures). * *P* < 0.05 vs. untreated control group (“Ctrl”). All in vitro experiments were repeated 3-4 times, and similar results were obtained.

To begin to test the efficacy of the DNA-PKcs/mTOR dual inhibitor CC-115 as a treatment for RCC, the established human RCC cell line, 786-O, was treated with gradually-increasing concentrations of CC-115 [11, 21]. Using the CCK-8 assay to test cell viability, we showed that CC-115 inhibited 786-O cell viability in a dose-dependent manner (Figure 1A). CC-115’s significant anti-survival activity was observed after 48-72h (Figure 1A). The IC-50 of CC-115, or the concentration resulting in 50% reduction of viability, was between 1-5 μM (48h and 72h treatment) (Figure 1A). Performing a soft agar colony formation assay, results confirmed that CC-115, at 1-10 μM, significantly decreased the number of viable 786-O cell colonies (Figure 1B). Furthermore, BrdU incorporation was suppressed after CC-115 (1-10 μM) treatment (Figure 1C). These results indicated a significant anti-proliferative activity by CC-115.

The potential activity of CC-115 on other RCC cells was studied. In both established (A489 cell line) and primary human RCC cells (derived from two patients, “RCC1/RCC2”), treatment with CC-115 (5 μM) for 48/72h significantly inhibited cell viability (CCK-8 optical density/OD, Figure 1D) and proliferation (BrdU ELISA OD, Figure 1E). Importantly, in the immortalized HK-2 human proximal tubule epithelial cells and primary human renal epithelial cells (“Pri-Epi”), CC-115 treatment (5 μM, 48/72h) was non-cytotoxic (Figure 1D) nor anti-proliferative (Figure 1E). These results indicated a cancer cell specific effect by the compound. Collectively, CC-115 potently inhibited RCC cell survival and proliferation.

### CC-115 induces apoptosis activation in human RCC cells

**Figure 2:**
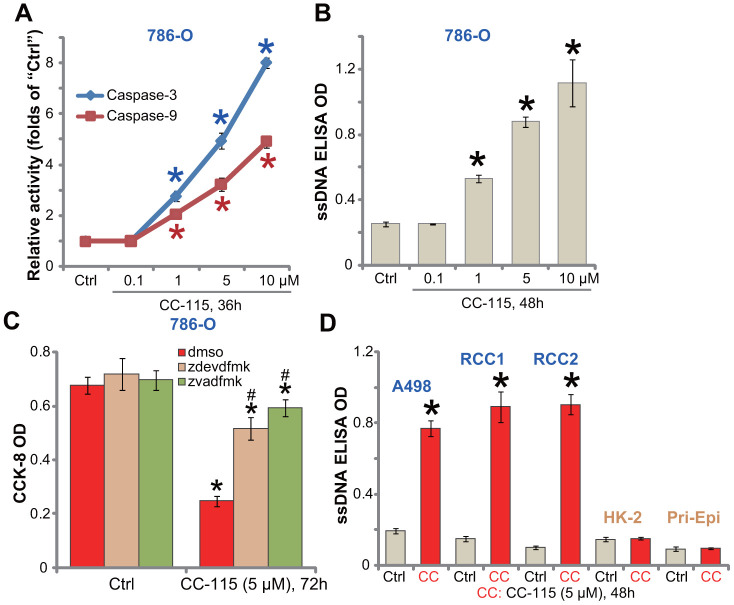
**CC-115 induces apoptosis activation in human RCC cells.** Established human RCC cell lines (786-O and A498), the primary human RCC cells (“RCC1/2”), HK-2 tubular epithelial cells and the primary human renal epithelial cells (“Pri-Epi”) were treated with indicated concentration of CC-115 for the applied time periods, cell apoptosis was tested by the assays mentioned in the text (**A**, **B**, **D**); For (**C**), 786-O cells were pretreated for 30 min with 50 μM of z-VAD-fmk (“zvadfmk”) or z-DEVD-fmk (“zdevdfmk”) before CC-115 treatment, and cell viability analyzed by CCK-8 assay. *P < 0.05 vs. untreated control group (“Ctrl”). ^#^ *P* < 0.05 vs. “dmso” (0.5% DMSO) (**C**).

Apoptosis activation is an important mechanism for reduced viability and proliferation of cancer cells. By analyzing caspase activity, we show that the caspase-3 and the caspase-9 activities were significantly increased in CC-115 (1-10 μM)-treated 786-O cells (Figure 2A). Furthermore, CC-115 resulted in single strand DNA (ssDNA) accumulation in 786-O cells, further confirming apoptosis activation (Figure 2B). The caspase-3 specific inhibitor z-DEVD-fmk and the pan caspase inhibitor z-VAD-fmk largely attenuated CC-115 (5 μM)-induced viability reduction in 786-O cells (Figure 2C). These results demonstrate that CC-115 induced apoptotic death in 786-O RCC cells.

In established A489 cells and primary RCC cells (“RCC1/RCC2”), treatment with CC-115 (5 μM) induced a significant increase of ssDNA content (Figure 2D), indicating apoptosis activation. On the contrary, CC-115 failed to induce significant apoptosis activation in HK-2 cells and primary human renal epithelial cells (“Pri-Epi”) (Figure 2D).

### CC-115 blocks DNA-PKcs and mTOR activation in RCC cells

**Figure 3:**
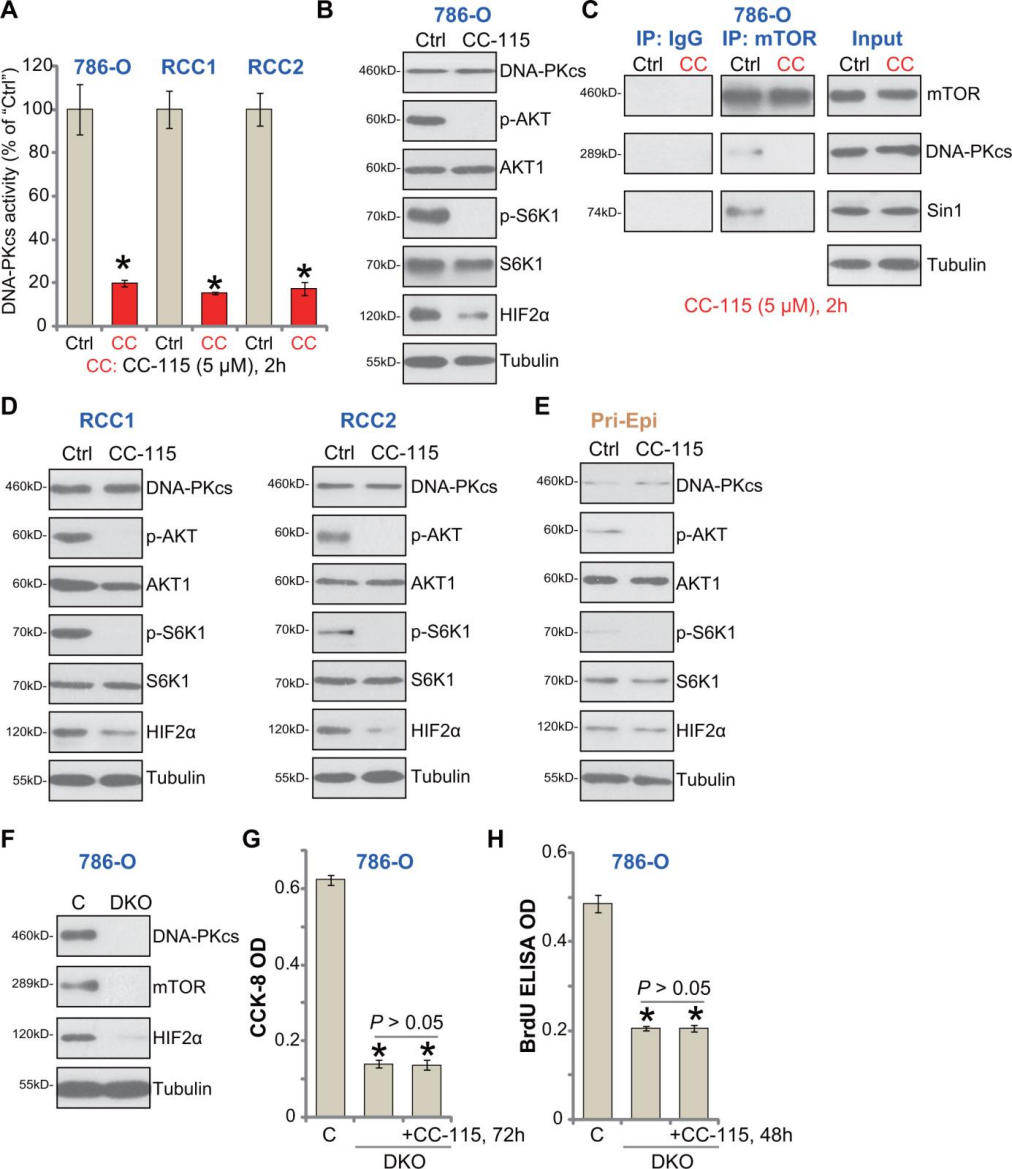
**CC-115 blocks DNA-PKcs and mTOR activation in RCC cells.** 786-O RCC cells, the primary human RCC cells (“RCC1/2”), or the primary human renal epithelial cells (“Pri-Epi”), were treated with CC-115 (“CC”, 5 μM) for 2h, DNA-PKcs activity (**A**) and expression of listed proteins in total cell lysates (**B**, **C**-”Inputs”, **D**, **E**) were shown. mTOR-Sin1-DNA-PKcs association was tested by co-immunoprecipitation assay (**C**, “IP”). Expression of the listed proteins in control 786-O cells (“C”) and DNA-PKcs- and mTOR-double knockout (DKO) 786-O cells were shown (**F**), cells were also treated with/without CC-115 (5 μM) for 48/72h, cell viability and proliferation were tested by CCK-8 assay (**G**) and BrdU ELISA assay (**H**), respectively. The exact same number of “C” and DKO cells were plated initially (“0h”) for the functional assays (**G**, **H**). *P < 0.05 vs. untreated control group (“Ctrl”) (**A**). *P < 0.05 vs. “C” cells (**G**, **H**).

To demonstrate that CC-115 acts as a DNA-PKcs and mTOR dual inhibitor, we tested its inhibitory effect on signaling pathways in RCC cells. When analyzing DNA-PKcs activity using a previously-described assay [22], we found that CC-115 treatment (5 μM, 2h) significantly inhibited DNA-PKcs activity in 786-O and primary RCC cells (“RCC1/2”) (Figure 3A). DNA-PKcs protein level was unchanged with CC-115 treatment (Figure 3B). The dual inhibitor blocked phosphorylation of S6K1 (Ser-389, the indicator of mTORC1 activation [3]) and AKT (Ser-473, the indicator of mTORC2 activation) in 786-O cells (Figure 3B). Furthermore, one mTORC2 target protein, HIF-2α [15, 23], was downregulated (Figure 3B).

Our previous study has shown that DNA-PKcs forms a complex with mTOR and Sin1, required for mTORC2 activation in RCC cells [15]. Performing a co-IP assay from 786-O cells, results demonstrated that CC-115 disrupted mTOR-Sin1-DNA-PKcs association (Figure 3C, “IP”), but did not inhibit their expression (Figure 3C, “Input”). In the primary human RCC cells (“RCC1/2”), CC-115 blocked AKT-S6K1 phosphorylation and downregulated HIF-2α (Figure 3D). Thus, CC-115 blocked DNA-PK and mTOR signaling in RCC cells.

In line with our previous findings [15], basal AKT-S6K1 phosphorylation as well as expression of DNA-PKcs and HIF-2α were extremely low in primary human renal epithelial cells (“Pri-Epi”) (Figure 3E). This could explain the ineffectiveness of the compound in normal epithelial cells (Figures 1, 2).

If DNA-PKcs-mTOR inhibition is the primary mechanism of CC-115-induced cytotoxicity in RCC cells, the compound should be ineffective in DNA-PKcs plus mTOR-depleted cells. To test this hypothesis, the lenti-CRISPR/Cas9 mTOR-KO construct and the lenti-CRISPR/Cas9 DNA-PKcs-KO construct were used to deplete expression in 786-O cells. Stable cells were established via FACS-mediated GFP sorting and puromycin selection. The immunoblotting confirmed DNA-PKcs and mTOR double knockout (DKO) (Figure 3F). When compared to the control cells (“C”), DKO cells presented with decreased cell viability (CCK-8 OD, Figure 3G) and proliferation (BrdU OD, Figure 3H). Importantly, adding CC-115 to the DKO 786-O cells failed to further inhibit cell viability and proliferation (Figure 3G, 3H). These results indicated that DNA-PKcs-mTOR dual inhibition is the reason of CC-115-induced RCC cell death and apoptosis.

In 786-O cells mTOR single KO, by CRISPR/Cas9-mediated gene editing (Supplementary Figure 1A), inhibited cell viability (Supplementary Figure 1B) and proliferation (BrdU incorporation, Supplementary Figure 1B). Importantly, in mTOR single KO cells adding CC-115 was able to induce further anti-786-O cell activity (Supplementary Figure 1B, 1C). Similarly, CRISPR/Cas9-induced DNA-PKcs single KO (Supplementary Figure 1D) only moderately inhibited 786-O cell viability (Supplementary Figure 1E) and proliferation (Supplementary Figure 1F), both were however augmented with CC-115 treatments (Supplementary Figure 1E, 1F). These results again confirmed that CC-115-induced anti-RCC cell activity was due to DNA-PKcs-mTOR dual inhibition.

### Autophagy inhibition sensitizes CC-115 in RCC cells

**Figure 4:**
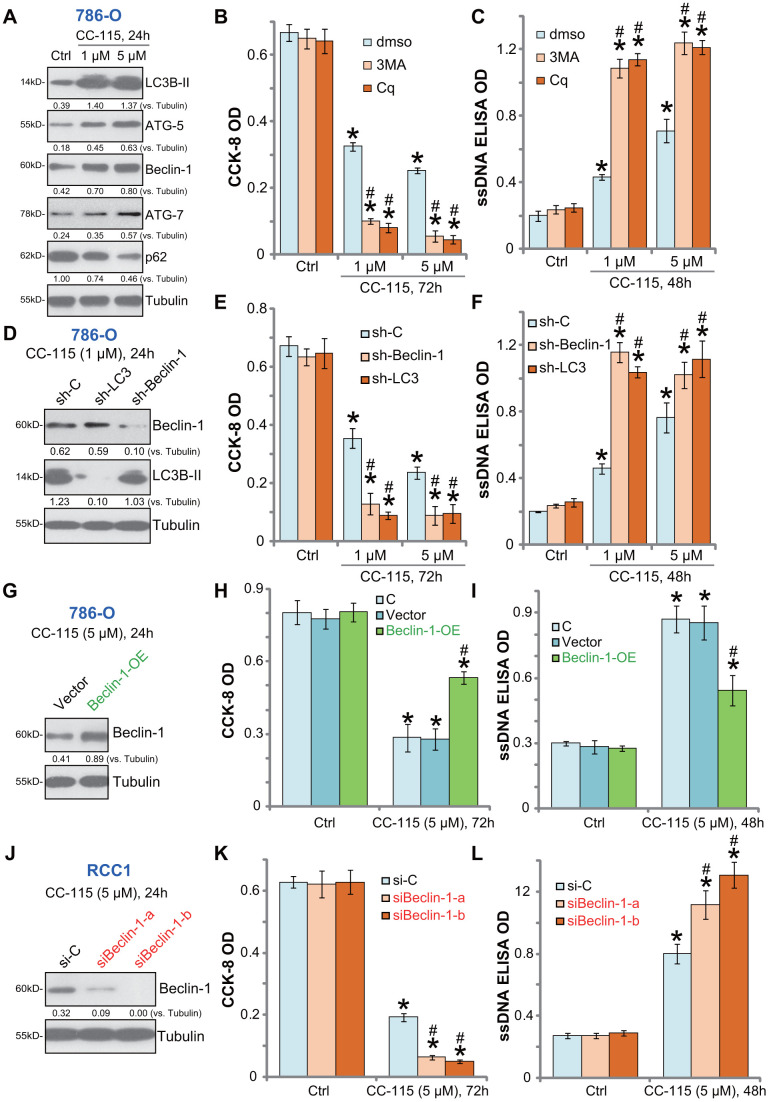
**Autophagy inhibition sensitizes CC-115 in RCC cells.** 786-O cells were treated with CC-115 (5 μM) for 24h, expression of listed proteins in total cell lysates were shown (**A**); 786-O cells were pre-treated with 3-methyladenine (3-MA, 1 mM), chloroquine (Cq, 5 μM) or 0.5% DMSO vehicle control (“dmso”) for 1h, followed by CC-115 (5 μM) treatment for indicated time periods, cell viability and apoptosis were tested by CCK-8 assay (**B**) and ssDNA ELISA assay (**C**), respectively. Stable 786-O cells with Beclin-1 shRNA (“sh-Beclin1”), LC3 shRNA (“sh-LC3”) or scramble control shRNA (“sh-C”), were treated with CC-115 (1/5 μM) for indicated time periods, expression of listed proteins (**D**), cell viability (**E**) and apoptosis (**F**) were tested. Vector control or the “Beclin-1-OE” 786-O cells were treated with/without CC-115 (5 μM) for applied time; Listed proteins were tested (**G**); Cell viability (**H**) and apoptosis (**I**) were tested similarly. Primary human RCC cells (“RCC-1”), transfected with Beclin-1 siRNA-a (“siBeclin1-a”, Santa Cruz Biotech), Beclin-1 siRNA-b (“siBeclin1-b”, Cellular Signaling Tech) or scramble control siRNA (“siCtrl”) (200 nM each), were treated with/without CC-115 (5 μM) for applied time; Listed proteins were tested by Western blot assay (**J**); Cell viability (**K**) and apoptosis (**L**) were tested. “C” stands for parental control cells (**H**, **I**). Expression of listed proteins were quantified and normalized to the loading control (**A**, **D**, **G**, **J**). *P < 0.05 vs. untreated control group (“Ctrl”). ^#^ *P* < 0.05 vs. “dmso” cells (**B**, **C**). ^#^ *P* < 0.05 vs. “sh-C”/”si-C” cells (**E**, **F**, **K**, **L**). ^#^ *P* < 0.05 vs. Vector control cells (**H**, **I**).

Our previous studies have shown that mTOR inhibition by AZD-2014 induced feedback autophagy activation, which counteracted RCC cell death/apoptosis. Whereas inhibition of autophagy sensitized AZD-2014-induced RCC cell activity [12]. In cancer cells, inhibition of DNA-PKcs could also induce autophagy activation as a negative feedback pro-survival factor [22]. In 786-O cells CC-115 induced autophagy activation, evidenced by light chain 3B (LC3B)-II induction, Beclin-1/ATG-5/ATG-7 upregulation and p62 degradation (Figure 4A). Pharmacologic autophagy inhibition by two well-established autophagy inhibitors, 3-MA and Cq [24, 25], significantly potentiated the CC-115-induced viability reduction (Figure 4B) and apoptosis (Figure 4C) in 786-O cells.

To exclude possible off-target effects of the applied autophagy inhibitors, the shRNA strategy was used to silence key autophagy proteins. As described, 786-O cells were transduced with lentivirus encoding Beclin-1 shRNA or LC3 shRNA, and stable cells established. Western blotting confirmed the silencing of targeted proteins by the specific lentiviral shRNA in cells with CC-115 treatment (Figure 4D). As shown CC-115-induced viability reduction (Figure 4E) and apoptosis activation (Figure 4F) were enhanced in Beclin-1-silenced or LC3-silenced 786-O cells. Therefore, autophagy inhibition sensitized 786-O cells to CC-115-induced cell death.

Beclin-1 is required for autophagosome formation [26, 27]. Ectopic overexpression of Beclin-1 will initiate autophagy [26]. A Beclin-1-overexpressing stable 786-O cell line (“Beclin-1-OE”) was established (Figure 4G). When compared to the vector control cells, CC-115-induced viability reduction (Figure 4H) and apoptosis (Figure 4I) were dramatically inhibited in Beclin-1-OE cells. In primary human RCC cells (“RCC-1”), transfection of “Beclin-1 siRNA-a” or “Beclin-1 siRNA-b” (see Methods) efficiently downregulated Beclin-1 (Figure 4J). Beclin-1-silenced cells were sensitized to CC-115-induced cytotoxicity (Figure 4K) and apoptosis (Figure 4L). These results confirmed the pro-survival function of autophagy activation in CC-115-treated RCC cells.

### CC-115 oral administration inhibits 786-O xenograft growth *in vivo*

**Figure 5:**
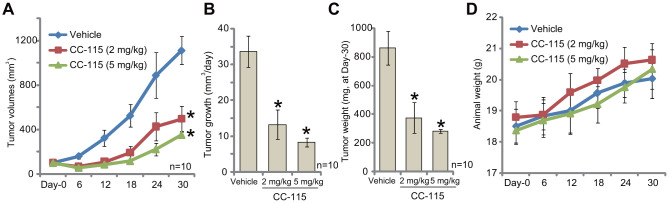
**CC-115 oral administration inhibits 786-O xenograft growth *in vivo*.** 786-O cells were inoculated via *s.c.* injection to the flanks of nude mice (five million cells per mouse). When tumors were close to 100 mm^3^ (“Day-0”), mice were randomly assigned into three groups, with ten mice per group. One group was treated with vehicle control (“Vehicle”) and the other two with CC-115 (2 and 5 mg/kg, daily, for 20 consecutive days). Tumor volumes (**A**) and estimated daily tumor growth (**B**) were shown. At Day-30, tumors of all three groups were isolated and weighted individually (**C**). Mice body weights were recorded (**D**). * *P* < 0.05 vs. “Vehicle” group. The mice experiments were performed once.

The *in vivo* activity of CC-115 was tested. As described [11, 12], 786-O cells were *s.c.* injected into the flanks of the nude mice. Xenografts were established within three weeks (tumor volumes close to 100 mm^3^, labeled as “Day-0”). The tumor-bearing mice were randomly assigned into three groups, receiving CC-115 or vehicle control. Results demonstrated that oral administration of CC-115, at 2 mg/kg or 5 mg/kg, significantly inhibited subcutaneous 786-O xenograft growth in nude mice (Figure 5A). The estimated daily 786-O tumor growth, calculated by (tumor volume at Day-30 subtracting tumor volume at Day-30)/30, was dramatically inhibited in CC-115-treated mice (Figure 5B). Tumors of all three groups were isolated at Day-30 and weighted (Figure 5C). Tumors with CC-115 treatment were much lighter than the vehicle-treated tumors (Figure 5C). The mouse body weights were not significantly different between the three groups (Figure 5D). Collectively, oral administration of CC-115 inhibited subcutaneous 786-O xenograft growth in mice.

## DISCUSSION

Our results demonstrated that CC-115, a DNA-PKcs/mTOR dual inhibitor, suppressed survival and proliferation in established (786-O and A489 lines) and primary human RCC cells. The dual inhibitor induced apoptosis activation in RCC cells. It was yet non-cytotoxic and pro-apoptotic to normal renal epithelial cells. In RCC cells CC-115 inhibited DNA-PKcs and mTORC1/2 signalings. *In vivo*, CC-115 oral administration at well-tolerated doses potently inhibited 786-O xenograft growth in nude mice. Therefore, dual inhibition of DNA-PKcs and mTOR by CC-115 potently inhibited RCC cell growth *in vitro* and *in vivo*.

We have previously shown that mTORC1/2 inhibition by AZD-2014 induced feedback pro-survival autophagy in RCC cells. Autophagy inhibition sensitized RCC cells to AZD-2014-induced anti-RCC cell activity [12]. DNA-PKcs activation could also inhibit autophagy activation [22]. Conversely, DNA-PKcs inhibition or silencing shall trigger pro-survival autophagy activation in RCC cells [22].

In RCC cells DNA-PKcs/mTOR dual inhibition by CC-115 induced autophagy activation, evidenced by Beclin-1/ATG-5/ATG-7 upregulation, LC3B-II induction and p62 degradation. Chloroquine (Cq) increases intra-lysosomal pH levels, thereby inhibiting autophagic protein degradation. Another autophagy inhibitor 3-MA prevents autophagosome formation via suppressing LC3B-I to LC3B-II conversion and autophagy induction [28]. Pretreatment with these two autophagy inhibitors potentiated CC-115-induced RCC cell death and apoptosis. These results demonstrated that autophagy is a key resistance mechanism to CC-115 in RCC cells. Furthermore, shRNA-mediated knockdown of Beclin-1 or LC-3 significantly enhanced CC-115-induced RCC cell death and apoptosis. Conversely, ectopic overexpression of Beclin-1 alleviated CC-115-induced cytotoxicity. These results further confirmed that feedback autophagy activation should be the key CC-115 resistance mechanism in RCC cells.

## CONCLUSIONS

Dual inhibition of mTOR and DNA-PKcs by CC-115 potently inhibits RCC cell growth, suggesting that this compound has important therapeutic value for RCC. Notably, studies have implied that some mTOR kinase inhibitors or the second generation of mTOR inhibitors failed to exert significant clinical improvement for RCC patients [6, 8]. Certain mTOR inhibitors are even more toxic and less efficacious than rapamycin and its analogs (everolimus or temsirolimus) [29]. Thus, the current results of CC-115 in RCC cells and mice RCC xenografts can’t be simply translated to humans. The pharmacokinetics, efficacy and safety of CC-115 in RCC also require further studies.

## MATERIALS AND METHODS

### Chemicals and antibodies

CC-115 was provided by MCE China (Shanghai, China). Puromycin, the caspase inhibitors z-DEVD-fmk and z-VAD-fmk as well as the autophagy inhibitors chloroquine (Cq) and 3-methyaldenine (3-MA) were obtained from Sigma-Aldrich (St. Louis, MO). The antibodies of this study were provided by Cell Signaling Tech (Shanghai, China). Cell culture reagents were purchase from Gibco (Suzhou, China).

### Cell culture

786-O and A498 RCC cells and HK-2 human proximal tubule epithelial cells were cultured as previously reported [12, 15]. The primary human RCC cells, derived from two different patients (“RCC1 and RCC-2”) as well as the primary human renal epithelial cells (“Pri-Epi”), were established previously [15, 30]. Primary human cells of passage 3-6 were utilized for *in vitro* experiments. The protocols were approved by the Ethical Committee of Nantong University (Nantong, China).

### Cell Counting Kit-8 (CCK-8) assay

Cells were seeded in 96-well tissue culture plates (at 3×10^3^ cells per well). Following treatments, CCK-8 (Dojindo, Kumamoto, Japan) assay was performed to quantify cell viability. CCK-8 optical densities (ODs) at 450 nm were recorded.

### Soft agar colony formation assay

Briefly, 786-O cells were plated onto 60 mm plates with 5×10^4^ cells per plate. After incubation with CC-115 for 10 days (CC-115 medium was renewed every two days), 786-O cell colonies were stained with 0.05% crystal violet, and manually counted.

### BrdU (5-bromo-2-deoxyuridine) ELISA assay

Following the applied treatments, the BrdU ELISA kit (Cell Signaling Tech) was employed to test cell growth *in vitro*. BrdU ELISA ODs at 450 nm were recorded.

### Single-stranded DNA (ssDNA) assay

Following the treatments, a ssDNA Apoptosis ELISA Kit (Chemicon International, Temecula, CA) was employed to quantify cell apoptosis via the attached protocol. ELISA ODs at 405 nm were recorded.

### Caspase activity assay

Caspase activity assay was performed as described [12, 30] The release of AFC was quantified by a Fluoroskan system with an excitation value of 355 nm and emission value of 525 nm.

### Western blotting

The detailed protocol of Western blotting assay was described as previously reported [12, 31]. Band intensity was quantified via the ImageJ software (NIH).

### Co-Immunoprecipitation (Co-IP)

As reported [12], from each treatment, aliquots of 1000 μg of protein (per treatment) were pre-cleared by adding protein A/G Sepharose (“Beads”, Sigma). The pre-cleared lysates were incubated with the specific anti-mTOR antibody overnight. Afterwards, protein A/G Sepharose were added to the lysates. Beads were washed and boiled, detected by Western blotting.

### shRNA and stable cell selection

For shRNA experiments, lentivirus were produced by constructing a lentiviral GV248 construct (Genechem, Shanghai, China) containing a puromycin resistance gene and either scramble control shRNA, or shRNA to Beclin-1 (“sh-Beclin-1”) or light chain 3 (LC3, “sh-LC3”). RCC cells were grown in six-well tissue culture plates (50×10^3^ cells per plate). Lentiviral-shRNA was added to the cells. Stable cells were selected by puromycin (5.0 μg/mL) for 10 days [32]. Expression of targeted protein in the stable cells was tested by Western blotting assay.

### DNA-PKcs and mTOR double knockout

The small guide RNAs (sgRNAs) for human *DNA-PKcs* (targeted DNA sequence, 5’-GATCAGTTGATGACCGGCCA) and *mTOR* (targeted DNA sequence, 5’-CAATACTGTTGAGTGACTTC) were synthesized by Genechem (Shanghai, China). Afterwards, the sgRNA was individually inserted into the lenti-CRISPR-GFP plasmid (Addgene, Beijing, China). mTOR and DNA-PKcs constructs were transfected to 786-O cells. Afterwards, GFP-positive single cells were FACS-sorted. Stable 786-O cells were achieved by further puromycin (5.0 μg/mL) selection for another week. In the stable cells, DNA-PKcs and mTOR double knockout (“DKO”) or single knockout (SKO) was confirmed by Western blotting.

### Beclin-1 siRNA

Two different Beclin-1 siRNAs were provided by Santa Cruz Biotech (sc-29797, labeled as “siBeclin-1-a”) and Cell Signaling Tech (#6222, labeled as “siBeclin-1-b”) [33]. The scramble control siRNA (labeled as “siCtrl”) was provided by Santa Cruz Biotech as well. Primary human RCC cells were initially seeded onto the 6-well tissue culture plates. Lipofectamine 2000 was utilized for siRNA (200 nM) transfection with the standard procedure. Beclin-1 knockdown was verified by Western blotting assay.

### Ectopic Beclin-1 overexpression

*Beclin-1 cDNA* inserted into the hU6-MCS-Ubiquitin-EGFP-IRES-puromycin vector (GV428 [34]) to generate *Beclin-1* construct. The latter and the lentivirus plasmids (Genepharm) were co-transfected to HEK-293 cells to generate viral particles. After filtration and enrichment (10^8^ TU/mL), virus were added to 786-O cells. Cells were cultured in puromycin (5 μg/mL) medium with 10-12 days. Beclin-1 overexpression was verified by Western blotting assay. Control cells were infected with virus with empty vector [34].

### Xenograft model

As reported [12], eight-week-old female nude/beige mice (4-5 week old, 17-18 g) were provided by Nantong University Animal Laboratories. For each mouse, five million 786-O cells (in 200 μL DMEM/Matrigel) were subcutaneously (*s.c.*) injected to the right flank. Xenograft tumors were established within three weeks when volume of each tumor was close to 100 mm^3^ (“Day-0”). Mice were then randomly assigned into three groups (n=10 of each group). Mice body weights and bi-dimensional tumor volumes were recorded every six days [12]. All experimental protocols were approved by the IACUC of Nantong University.

### Statistical analyses

Data were expressed as mean ± standard deviation (SD). Statistical analyses were performed as described [11, 30]. *P* values of <0.05 were considered statistically significant.

## Supplementary Material

Supplementary Figure 1
