# Supplementary material for: Dual inhibition of DNA-PKcs and mTOR by CC-115 potently inhibits human renal cell carcinoma cell growth
Source: Aging (Albany NY). 2020 Oct 27;12(20):20445–56. doi: 10.18632/aging.103847 (PMC7655216; doi:10.18632/aging.103847)
Supplement: Supplementary Figure 1 [file aging-12-103847-s001..pdf]

SUPPLEMENTARY FIGURE

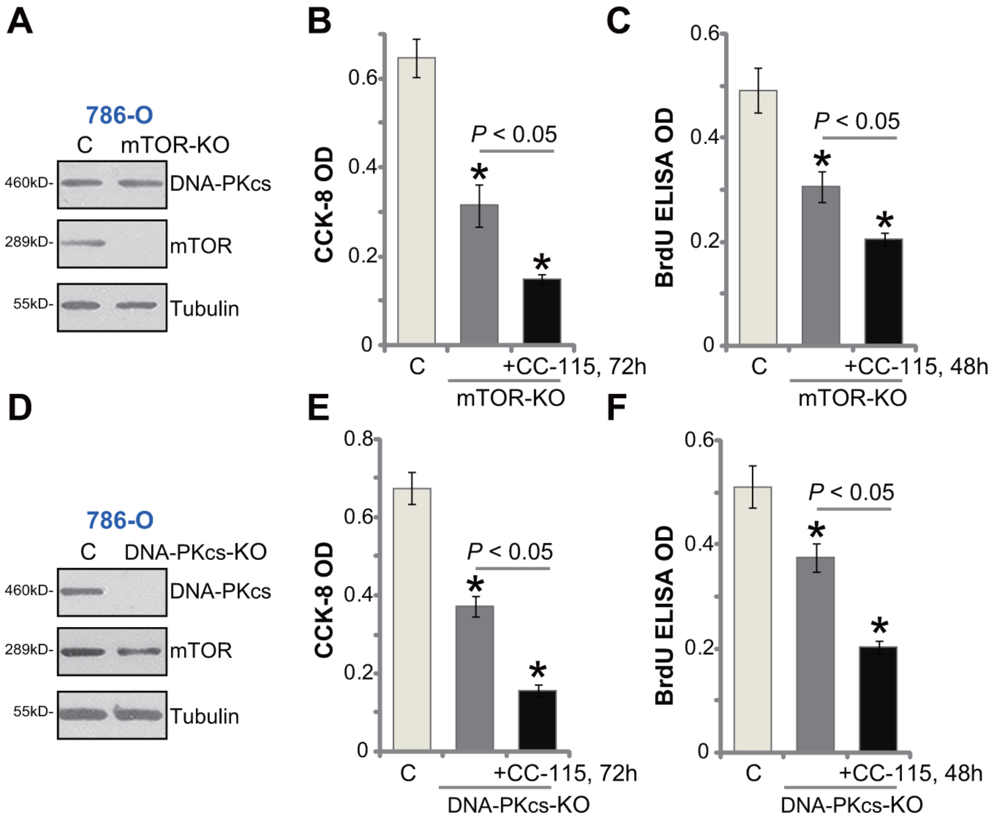

Supplementary Figure 1. Expression of the listed proteins in control 786-O cells (“C”), mTOR-single knockout (mTOR-KO) or DNA-PKcs-single knockout (DNA-PKcs-KO) 786-O cells were shown (**A**, **D**); The single knockout cells were treated with or without CC-115 (5 µM) for 48-72h, cell viability and proliferation were tested by CCK-8 assay (**B**, **E**) and BrdU ELISA assay (**C**, **F**), respectively. The exact same number of “C” and single knockout 786-O cells were plated initially (“0h”) for the functional assays. *$P < 0.05$ vs. “C” cells.
